# Engineering Hydrogels with Enhanced Adhesive Strength Through Optimization of Poly(Ethylene Glycol) Molecular Weight

**DOI:** 10.3390/polym17050589

**Published:** 2025-02-23

**Authors:** Yin-An Yang, Yu-Feng Ni, Rajan Deepan Chakravarthy, Karl Wu, Mei-Yu Yeh, Hsin-Chieh Lin

**Affiliations:** 1 Department of Chemistry, Chung Yuan Christian University, Taoyuan City 320314, Taiwan; a0976757006@gmail.com (Y.-A.Y.); ricky86831@gmail.com (Y.-F.N.); 2 Department of Materials Science and Engineering, National Yang Ming Chiao Tung University, Hsinchu 300093, Taiwan; deepannycu@gmail.com; 3 Department of Orthopaedic Surgery, Far Eastern Memorial Hospital, New Taipei City 220216, Taiwan; 4 Center for Intelligent Drug Systems and Smart Bio-Devices (IDS2B), National Yang Ming Chiao Tung University, Hsinchu 30068, Taiwan

**Keywords:** poly(ethylene glycol), acrylamide, cellulose, adhesive, hydrogel

## Abstract

Hydrogels are extensively utilized in biomedical fields because of their remarkable properties, including biocompatibility, high water content, flexibility, and elasticity. However, despite substantial progress in hydrogel research, creating a hydrogel adhesive that integrates high stretchability, fatigue resistance, and reversible adhesion continues to pose significant challenges. In this study, we aimed to address these challenges by preparing hydrogels using a combination of acrylic acid, acrylamide, carboxymethylcellulose methacrylate, thiol-functionalized polyhedral oligomeric silsesquioxane, and poly(ethylene glycol) dimethacrylate (PEGDM). By systematically varying the molecular weight of PEG, we were able to precisely adjust the mechanical and adhesive properties of the hydrogels. Our research revealed that a PEG molecular weight of 2000 (resulting in P1 hydrogel) provided a notable adhesive strength of 717.2 kPa on glass surfaces. This performance is particularly impressive given the challenges associated with achieving high adhesive strength while maintaining other desirable hydrogel properties. Beyond its strong adhesive capabilities, the P1 hydrogel also demonstrated exceptional stretchability, support, and fatigue resistance. These characteristics are crucial for applications where the adhesive needs to endure repeated stress and deformation without losing effectiveness. The successful development of P1 hydrogel underscores its potential as a multifunctional adhesive material with a broad range of applications. The ability to tailor the properties of hydrogels through molecular weight adjustments offers a promising approach to creating advanced adhesive solutions that meet the demanding requirements of modern biomedical and industrial applications.

## 1. Introduction

Hydrogels are three-dimensional (3D) polymer networks that can be physically or chemically cross-linked, capable of retaining water while preserving a distinctive 3D structure, similar to that of natural tissues, such as cartilage, skin, muscle, and the extracellular matrix [1,2]. Their semi-solid mechanical properties and fluid-like transport capabilities make hydrogels promising materials for applications in biomedicine, electronic skins, and strain sensors, while they have also been utilized in cosmetology as injection implants [3,4,5]. A specific type of hydrogel, known as hydrogel adhesives, can bond both inorganic and organic materials, making them ideal for use in wound closure substrates, cartilage repair matrices, and other medical applications [6,7,8,9]. Furthermore, hydrogel adhesives are increasingly utilized in electronic and industrial sectors, where their combination of mechanical strength and adhesive qualities provides innovative solutions for challenges such as sealing and bonding in flexible electronics and soft robotics [10,11,12]. For example, Liu and Gu, inspired by the dynamic reversibility of interactions such as metal coordination and Schiff base bonds, developed high-performance hydrogel sensors with repeatable adhesion and self-healing capabilities [13]. They designed a multifunctional, polysaccharide-based dual-network conductive hydrogel sensor called DCMC/CS/PAA (DCP). This sensor features a primary network composed of chitosan (CS) and dialdehyde carboxymethyl cellulose (DCMC) linked through Schiff base reactions, and a secondary network formed by acrylic acid (AA) chains and aluminum ions. The coordination among polysaccharides in this network imparts multifunctional properties to the hydrogel. The DCP hydrogel contains numerous carboxyl and amino groups, which contribute to its strong adhesion to various substrates. Additionally, the formation of Schiff base bonds between the amino groups in the substrate and the aldehyde groups in the hydrogel enhances adhesion further. Despite these significant advancements in multifunctional hydrogels, developing a hydrogel adhesive that combines high stretchability, fatigue resistance, and reversible adhesion continues to be a challenging task.

Acrylic acid (AA) and acrylamide (AAM) are commonly used substances for synthesizing and preparing hydrogels. Polyacrylic acid (PAA) and polyacrylamide (PAAM) are extensively utilized in biomedical fields because of their non-toxic, biocompatible, and biodegradable characteristics. These polymers find applications in tissue engineering, wound healing, drug delivery, antimicrobial treatments, and cancer theranostics [14,15,16,17]. However, hydrogels based on AA and AAM may encounter issues such as reduced gel strength, dispersibility, and elasticity after absorbing water. They may also suffer from structural inhomogeneity and decreased mechanical strength due to a lack of hydrolytic stability [18]. To address these challenges, in this study, we incorporated carboxymethyl cellulose (CMC), polyhedral oligomeric silsesquioxane (POSS), and poly(ethylene glycol) (PEG) to enhance the functionality of the hydrogel [19,20,21]. These compounds are known for their ability to improve the mechanical, thermal, and surface properties of composite materials, provide excellent antioxidant properties, and reduce inflammatory responses [19,20,21]. In order to achieve strong bonding of these polymers with AA and AAM, we chemically modified them into carboxymethylcellulose methacrylate (CMC-MA), thiol-functionalized POSS-8SH, and poly(ethylene glycol) dimethacrylate (PEGDM) [22,23,24]. In our systematic investigation, we explored the impact of varying molecular weights of PEGDM on the mechanical and adhesive properties of hydrogels. Our findings revealed that PEG with a molecular weight of 2000 exhibited a notable adhesive strength of 717.2 kPa on glass, which is significant for applications requiring strong adhesion. While this hydrogel formulation did not exhibit the highest mechanical properties compared to others in the study, it did present an optimal balance between tensile strength, support, and fatigue resistance. This balance is crucial for practical applications where hydrogels must withstand repeated mechanical stress without significant degradation in performance. The successful development of this hydrogel highlights the importance of optimizing molecular weight to enhance specific properties like adhesion and durability.

## 2. Materials and Methods

### 2.1. Materials

Carboxymethyl cellulose (CMC, with a molecular weight of 250,000 g/mol and a degree of substitution of 0.7) and methacrylic anhydride were sourced from Sigma-Aldrich (St. Louis, MO, USA). Acrylamide (AAM) was acquired from Acros Organics (Geel, Belgium). N,N′-methylenebisacrylamide (MBAA), acrylic acid (AA), and poly(ethylene glycol) (PEG) with molecular weights of 2000 g/mol, 4000 g/mol, 8000 g/mol, 12,000 g/mol, and 20,000 g/mol were obtained from Alfa Aesar (Haverhill, MA, USA). 4-Dimethylaminopyridine, (3-mercaptopropyl) trimethoxysilane (MPTS), and lithium phenyl (2,4,6-trimethylbenzoyl) phosphinate (LAP) were purchased from Matrix Scientific (Elgin, SC, USA), Thermo Scientific (Waltham, MA, USA), and TCI (Tokyo, Japan), respectively. Deionized water was used in all experiments.

### 2.2. Synthesis of CMC-MA

According to the literature method [22], dissolve carboxymethyl cellulose (2.00 g) and 4-dimethylaminopyridine (0.93 g) in 45 mL of deionized water by heating and stirring the mixture at 90 °C until all solids are dissolved. After cooling the solution, methacrylic anhydride (1.32 mL) was added while the solution was kept in an ice bath. The mixture was stirred in the ice bath for 24 h. Subsequently, the solution was dialyzed using a dialysis membrane at 40 °C for 3 days and then lyophilized for 3 days to yield solid CMC-MA with a 48% yield.

### 2.3. Synthesis of POSS-8SH

According to the synthesis procedure [23], dissolve 10 mL of MPTS in 240 mL of methanol and add 20 mL of concentrated hydrochloric acid. Stir and reflux the mixture at 90 °C for 24 h. After allowing the solution to cool, white precipitates will form. Wash these precipitates three times with cold methanol, then dissolve them in dichloromethane (DCM). Extract the DCM solution with deionized water three times. Separate the organic phase, dry it using anhydrous MgSO_4_, and remove the dichloromethane under vacuum to obtain POSS-8SH with a yield of 53%. HRMS (ESI) *m*/*z* for C_24_H_56_O_12_S_8_Si_8_ [M]^+^, calcd 1017.8600, found 1017.8219.

### 2.4. Synthesis of PEGDM

According to the literature method [24], combine PEG (2.00 g, 1.00 mmol) (molecular weight: 2000 g/mol) and methacrylic anhydride (1.49 mL, 10 mmol) in a reaction flask. React at 150 °C for 10 min, then add ethyl ether and stir in an ice bath for 1 h to precipitate the product. Filter and collect the solid product, then dry it under vacuum to obtain PEG_2000_DM (yield: 41%). PEG_4000_DM (yield: 59%), PEG_8000_DM (yield: 63%), PEG_12000_DM (yield: 60%), and PEG_20000_DM (yield: 66%) were synthesized by combining PEG (1.00 mmol) (molecular weights: 4000 g/mol, 8000 g/mol, 12,000 g/mol, and 20,000 g/mol, respectively) with methacrylic anhydride (1.49 mL, 10 mmol) in a reaction flask. The reaction was carried out at 150 °C for 10 min, followed by the addition of ethyl ether. The mixture was then stirred in an ice bath for 1 h to precipitate the product.

### 2.5. Preparation of Hydrogels P0 to P1

Dissolve 50 mg of POSS-8H in 2.5 mL of AA (0.71% *v*/*v*), then add AAM (280 mg), CMC-MA (2.14% *w*/*v*), and MBAA (1.40% *w*/*v*) in 1.4 mL of deionized water. After that, add the photoinitiator LAP (0.7% *w*/*v*) and stir until fully dissolved. Expose the mixture to the UV oven (UV power: 1000 W, temperature: 60 °C) for 5 min to form the gel of P0. To prepare P1, dissolve 50 mg of POSS-8H in 2.5 mL of AA (0.71% *v*/*v*), then add PEG_2000_DM (0.05 mmol), AAM (280 mg), CMC-MA (2.14% *w*/*v*), and MBAA (1.40% *w*/*v*) in 1.4 mL of deionized water. After that, add the photoinitiator LAP (0.7% *w*/*v*) and stir until fully dissolved. Expose the mixture to the UV oven (UV power: 1000 W, temperature: 60 °C) for 5 min to form the gel. P2, P3, P4, and P5 were prepared by dissolving 50 mg of POSS-8H in 2.5 mL of AA (0.71% *v*/*v*), then adding AAM (280 mg), CMC-MA (2.14% *w*/*v*), MBAA (1.40% *w*/*v*), and either PEG_4000_DM (0.05 mmol), PEG_8000_DM (0.05 mmol), PEG_12000_DM (0.05 mmol), or PEG_20000_DM (0.05 mmol) into 1.4 mL of deionized water, respectively.

### 2.6. Measurements

Nuclear magnetic resonance (NMR) spectra were obtained using a Bruker (Billerica, MA, USA) AVANCEII-400 MHz spectrometer, with D_2_O or CDCl_3_ as solvents. The hydrogels were prepared using a UV oven, OPAS (Taichung, Taiwan) XLite 600. Mechanical testing was conducted on a Gotech (Taichung, Taiwan) AI-3000-U tensile tester at a stretching speed of 100 mm/min (sample dimensions: 15 mm × 10 mm × 2 mm) [25]. The adhesive strength of the hydrogels was evaluated on substrates of glass, Al, or Cu using a lap shear test at a speed of 10 mm/min (sample dimensions: 20 mm × 20 mm × 0.5 mm) [25]. Toughness was analyzed by calculating the area under the stress-strain curve obtained from a tensile test. This area represents the material’s ability to absorb energy before failure, providing a quantitative measure of toughness, which reflects both its strength and ductility under applied stress [26]. Molecular characteristics and interactions of the hydrogels were assessed using a Thermo Fisher Scientific (Waltham, MA, USA) Nicolet iS5 Fourier-transform infrared (FT-IR) spectrometer.

## 3. Results and Discussion

### 3.1. Hydrogel Preparation

**Figure 1:**
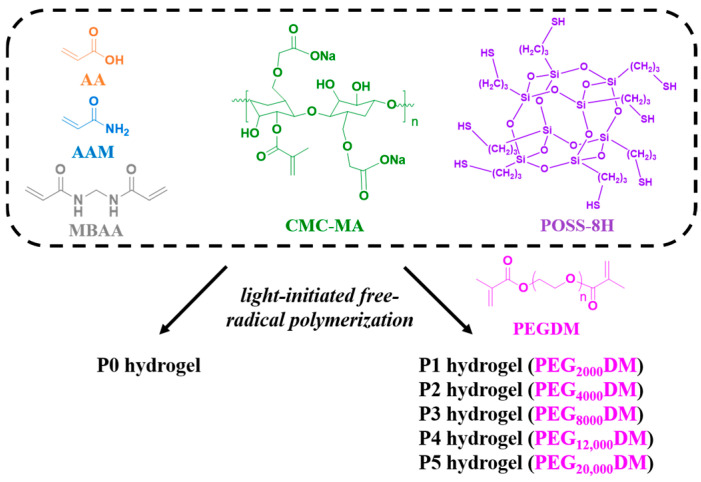
Schematic diagram of P0–P5 hydrogels.

As shown in Figure 1, we designed a covalently bonded hydrogel scaffold by carrying out free radical polymerization of AA, AAM, CMC-MA, PEGDM, and MBAA. The inclusion of POSS-8SH significantly enhanced the structural integrity and functionality of the scaffold. POSS-8H improved the mechanical properties of the hydrogel while also enabling additional covalent and non-covalent interactions, such as Michael addition reactions and hydrogen bonding [27,28]. This dual functionality not only strengthens the hydrogel’s framework but also provides opportunities for further chemical modification or functionalization, thereby greatly enhancing the material’s overall performance and adaptability. Moreover, PEGDMA is a hydrogel capable of absorbing large amounts of water, and the swelling behavior of water-responsive and shape-reconfigurable adhesives can be controlled by adjusting the molecular weight or concentration of PEGDMA in solvents such as water [29]. Therefore, the hydrogels investigated in this study were categorized based on the molecular weight of the PEG monomer used. The base hydrogel, lacking PEGDM, was termed P0. Subsequent variations, P1 through P5, were synthesized using PEGDM with molecular weights of 2000, 4000, 8000, 12,000, and 20,000, respectively. This systematic variation in PEG molecular weight is a crucial aspect of our research, as it enables the precise tuning of the hydrogel’s adhesive and mechanical properties.

### 3.2. The Tensile and Adhesive Strength of Hydrogels

**Figure 2:**
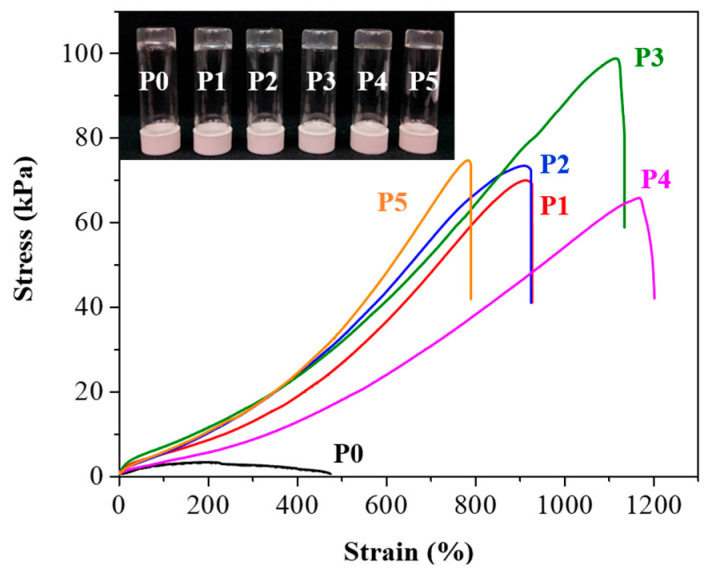
Tensile stress-strain curves of P0–P5 hydrogels. Black for P0, red for P1, blue for P2, green for P3, magenta for P4, and orange for P5. Inset: optical images of the hydrogels, arranged from left to right, are P0, P1, P2, P3, P4, and P5.

To create the P0 hydrogel, we combined AA, AAM, CMC-MA, POSS-8SH, and MBAA with LAP as the photoinitiator [30]. The synthesis process utilized light-initiated free-radical polymerization, a technique that generates free radicals from the initiator under light exposure, thereby initiating polymer chain formation [31]. To investigate the effect of different polymer chain lengths on the hydrogel properties, we introduced PEG monomers with varying molecular weights—PEG_2000_DM, PEG_4000_DM, PEG_8000_DM, PEG_12000_DM, and PEG_20000_DM—resulting in a series of hydrogels named P1 through P5. The variation in molecular weights was designed to explore the influence of polymer chain length on properties such as mechanical strength, elasticity, and adhesion. PEG is well-known for its biocompatibility and ability to modify the mechanical properties of hydrogels, making it an appropriate choice for our experiments [32]. As shown in Figure 2, the P0–P5 hydrogels exhibited a transparent appearance, indicative of uniform polymerization and successful incorporation of PEG monomers. The mechanical properties of the P0–P5 hydrogels are illustrated in Figure 2 and summarized in Table S1. The stress values observed in these hydrogels varied significantly, with P0 showing the lowest stress value at 1.4 kPa and P1 to P5 demonstrating higher stress values. This increase in stress values with the introduction of PEG monomers suggests that the presence of PEG chains enhances the mechanical strength of the hydrogel network. The specific stress values for P1, P2, P3, P4, and P5 were 69.6 kPa, 73.2 kPa, 98.9 kPa, 65.9 kPa, and 74.8 kPa, respectively, indicating that the mechanical properties can be finely tuned by adjusting the molecular weight of the PEG monomers used. Strain values also varied across the hydrogels, with P0 exhibiting a strain of 451%, and the PEG-modified hydrogels showing significantly higher strain values. P1, P2, P3, and P4 had strain values of 920%, 918%, 1120%, and 1172%, respectively, while P5 showed a strain of 786%. The incorporation of PEG with appropriate molecular weight increases the mechanical properties of the hydrogel. As the molecular weight of PEG increases from 2000 to 8000, the hydrogel’s elasticity and flexibility gradually improve. However, when the PEG molecular weight exceeds 12,000, there is a significant reduction in both the stress and strain of the hydrogel. To further investigate this phenomenon, we performed swelling experiments on P1 to P5. As shown in Figure S1, the swelling ratio of the hydrogel significantly increases when the PEG molecular weight exceeds 12,000, which seems to correlate with our observation that the mechanical properties of the hydrogel change as the PEG molecular weight increases [33].

**Figure 3:**
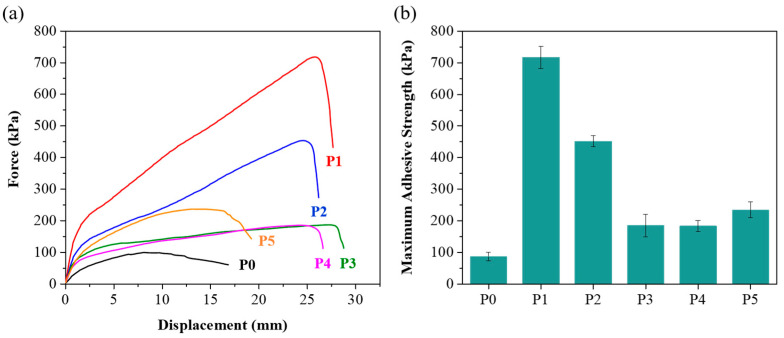
(**a**) Lap-shear strength test of P0–P5 hydrogels. Black for P0, red for P1, blue for P2, green for P3, magenta for P4, and orange for P5. (**b**) Maximum adhesive strength of P0–P5 hydrogels.

In addition to mechanical properties, adhesion plays a crucial role in hydrogel performance. It is known that achieving good adhesion on rough surfaces is relatively easier, while maintaining high adhesion on smooth surfaces such as glass presents a significant challenge [34]. The adhesion values on glass substrates further distinguished the hydrogels (Figure 3). The unmodified P0 hydrogel had an adhesion value of 87.6 kPa, while the PEG-modified hydrogels demonstrated significantly higher adhesion values. P1 exhibited the highest adhesion at 717.2 kPa, which is substantially greater than that of the other hydrogels. P2, P3, P4, and P5 showed adhesion values of 455.2 kPa, 184.9 kPa, 186.6 kPa, and 231.4 kPa, respectively. The experimental results indicate that higher PEG molecular weights are associated with lower adhesion strength, likely due to the enhanced hydrophilicity of higher molecular weight PEG [35]. While PEG with lower molecular weight improves adhesion to substrates, hydrogels prepared with PEG molecular weights below 2000 are generally brittle and lack stretchability. Notably, compared to the adhesion values reported in other studies (Table S2), the materials developed in this work show a substantial improvement in adhesion strength on glass. This advancement addresses the difficulty of securing strong adhesion on smooth surfaces. Moreover, higher adhesion in hydrogels like P1 could be advantageous in biomedical applications where strong attachment to tissues or other surfaces is required.

### 3.3. Biocompatibility of Hydrogels

**Figure 4:**
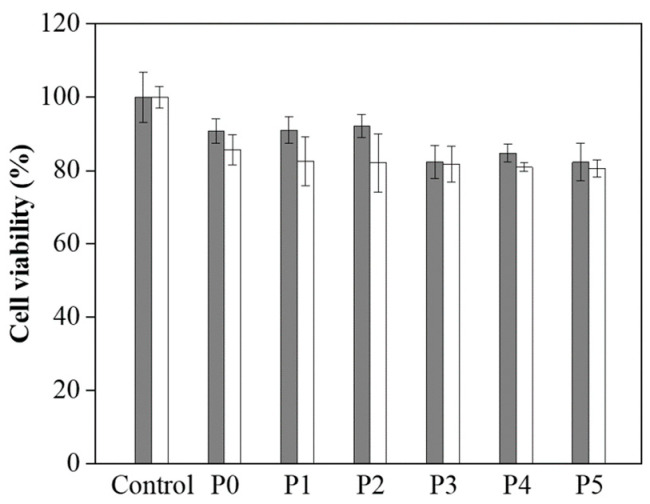
Cell viability of L929 cells on P0–P5 hydrogels after 1 day (grey) and 3 days (white).

Along with assessing the mechanical properties of the P0–P5 hydrogels, we also performed biocompatibility testing to investigate their potential for biomedical applications. The MTT (3-[4,5-dimethylthiazol-2-yl]-2,5-diphenyltetrazolium bromide) assay was employed to assess the compatibility of these hydrogels with living cells, specifically L929 fibroblast cells [36,37]. This assay measures cellular metabolic activity as an indicator of cell viability, proliferation, and cytotoxicity, making it a reliable method for evaluating biocompatibility [38,39]. The results, illustrated in Figure 4, show that the L929 cells maintained viability on the P0–P5 hydrogels over 1 and 3 days. Notably, the cell survival rate was approximately 85% after 3 days of culture, suggesting that the hydrogels provide a conducive environment for cell growth and proliferation. This level of biocompatibility indicates that the hydrogels are suitable for prolonged contact with living tissues [38,39]. The high cell viability rate observed across all samples (P0–P5) highlights their non-cytotoxic nature and reinforces their potential for a wide range of biomedical applications.

### 3.4. Hydrogel Analysis

**Figure 5:**
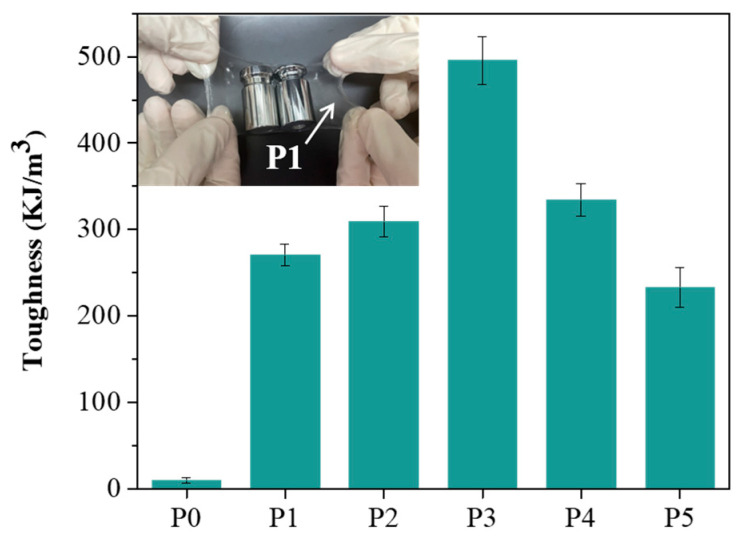
Toughness of P0–P5 hydrogels. Inset: Image of the P1 hydrogel carrying a 200 g weight.

Next, we analyzed the toughness of P0 to P5, which were found to be 9.8, 270.6, 309.4, 495.5, 334.3, and 233.1 kJ/m^3^, respectively, as shown in Figure 5. We observed that as the molecular weight of PEG increased from 2000 to 8000, the toughness gradually increased, reaching a peak at a molecular weight of 8000. However, when the molecular weight was further increased to 12,000, the toughness decreased. This decrease is likely due to the increased water solubility of high molecular weight PEG, which weakens the mechanical properties of the hydrogel, thereby affecting its toughness. As the molecular weight of PEG increases, the polymer chains become more hydrated and less tightly bound, leading to a reduction in the structural integrity of the hydrogel network. This disruption in the crosslinking structure results in a decrease in the hydrogel’s ability to withstand deformation, thus lowering its toughness [35]. Additionally, the increased solubility of higher molecular weight PEG in water can cause the hydrogel to become more susceptible to swelling, further compromising its mechanical performance [29]. These findings emphasize the delicate balance between molecular weight and hydrogel properties, where a higher molecular weight can enhance flexibility and stretchability but may also negatively impact the material’s overall strength and toughness. Based on the above experimental results, although P1 does not have the best mechanical performance among this series of hydrogels, it exhibits excellent adhesion properties. To evaluate its support capacity, we placed a 200 g weight on the P1 hydrogel, and the test confirmed that the P1 hydrogel has sufficient support capacity (Figure 5).

**Figure 6:**
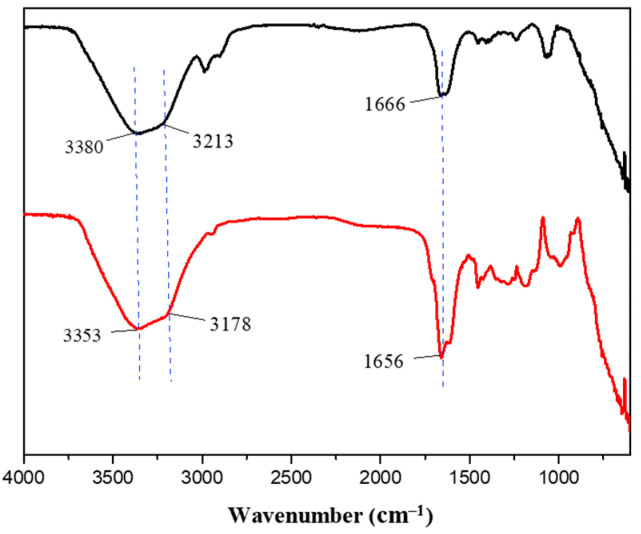
FT-IR spectra of P0 (black) and P1 (red) hydrogels.

Given the observed improvements in tensile strength and adhesion of the P1 hydrogel compared to P0, we sought to understand the molecular interactions underlying these enhancements. To do so, we conducted Fourier-transform infrared spectroscopy (FT-IR) analysis on both P0 and P1 hydrogels. As presented in Figure 6, the FT-IR spectrum of the P0 hydrogel displayed characteristic peaks at 3380 cm^−1^ and 3212 cm^−1^, which correspond to the -OH and -NH groups, respectively [40,41]. In addition, a peak at 1666 cm^−1^ was observed, indicative of C=O stretching vibrations [42]. These functional groups are integral to the polymer network structure, contributing to the hydrogel’s baseline physical properties. Upon analysis of the P1 hydrogel, the FT-IR spectra showed corresponding peaks at slightly shifted positions: 3353 cm^−1^, 3178 cm^−1^, and 1656 cm^−1^, respectively. These shifts towards lower wavenumbers suggest the formation of new intermolecular interactions, particularly hydrogen bonds, as a result of introducing PEGDM into the hydrogel matrix [43,44,45]. The presence of PEGDM likely enhances the network density and crosslinking within the hydrogel, which can significantly alter its mechanical and adhesive properties. The formation of hydrogen bonds can increase the cohesion within the hydrogel network, contributing to its improved tensile strength and elasticity. This is because hydrogen bonds can act as reversible physical crosslinks, which distribute stress more evenly throughout the material when it is stretched or deformed [46,47,48]. Furthermore, these bonds can help the hydrogel maintain its integrity and recover its shape after being subjected to mechanical stress, which is crucial for applications requiring repeated use or exposure to dynamic forces.

### 3.5. Evaluation of Hydrogel Performance in Practical Applications

**Figure 7:**
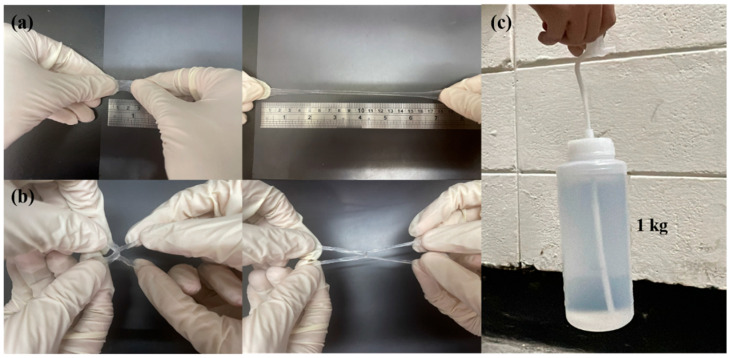
Tensile property test of P1 hydrogel: (**a**) before (left) and after (right) lateral stretching, (**b**) before (left) and after (right) cross stretching, and (**c**) lifting a 1 kg weight.

**Figure 8:**
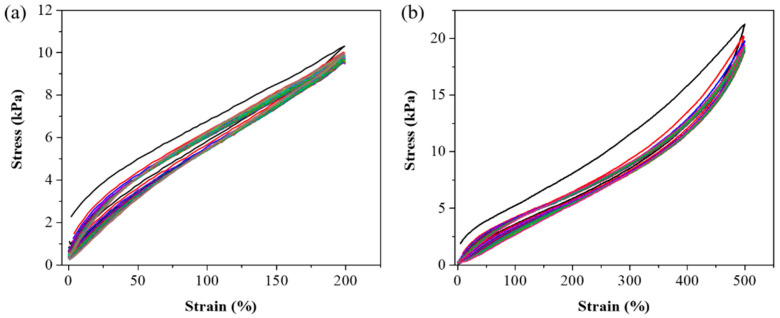
Loading-unloading cyclic curves of P1 hydrogel. (**a**) 20 cycles at 200% strain, and (**b**) 15 cycles at 500% strain.

After completing mechanical measurements, adhesive testing, biocompatibility assessments, and FT-IR analysis, we selected the P1 hydrogel for further experimental validation to explore its practical applications. The P1 hydrogel was subjected to various mechanical tests to evaluate its strength and durability. As illustrated in Figure 7a, the hydrogel exhibited remarkable elasticity, capable of being stretched laterally to 18 times its original length without breaking. This high level of stretchability indicates that the hydrogel can undergo significant deformation, which is crucial for applications requiring flexible and resilient materials. Further tests in Figure 7b involved cross-stretching the hydrogel, highlighting its ability to endure stress even when stretched with a smaller contact area. This characteristic suggests that the hydrogel can maintain its integrity and functionality under different mechanical conditions, which is valuable for applications where the material may experience various forms of mechanical stress. In addition to its elasticity and strength, Figure 7c showcases the hydrogel’s impressive load-bearing capacity, as it was able to lift a 1 kg water bottle without structural failure. This ability to support substantial weight relative to its own mass underscores the hydrogel’s potential for use in load-bearing biomedical applications. To further investigate the durability and resilience of the P1 hydrogel, we conducted fatigue resistance testing using a tensile testing machine [49,50]. This involved repeated loading-unloading cycles to evaluate the hydrogel’s ability to withstand mechanical stress over time. The stress-strain curves obtained from these cyclic tests, as illustrated in Figure 8, revealed the presence of hysteresis loops. Specifically, the hydrogel was subjected to deformation levels of 200% and 500%, simulating substantial mechanical strain. The first cycle exhibited a more pronounced hysteresis loop, which is typically expected as the material undergoes initial structural adjustments. Figures S2 and S3 indicate the energy dissipation of the P1 hydrogel. Compared to the second cycle, the energy dissipation in each subsequent cycle was reduced by approximately 9% or less over 20 cycles at 200% strain, and by about 5% or less over 15 cycles at 500% strain, suggesting excellent energy retention and mechanical stability. This behavior demonstrates the hydrogel’s impressive ability to return to its original state, highlighting its potential for applications that require materials capable of enduring repetitive mechanical stress without significant degradation. Meanwhile, the minimal change in the hysteresis loop area after the first cycle also indicates low internal friction and energy loss, attributes that are advantageous in applications such as soft robotics, flexible electronics, and biomedical devices, where long-term mechanical performance and reliability are crucial. These findings underscore the P1 hydrogel’s potential as a robust and durable material, capable of maintaining functionality in dynamic environments.

**Figure 9:**
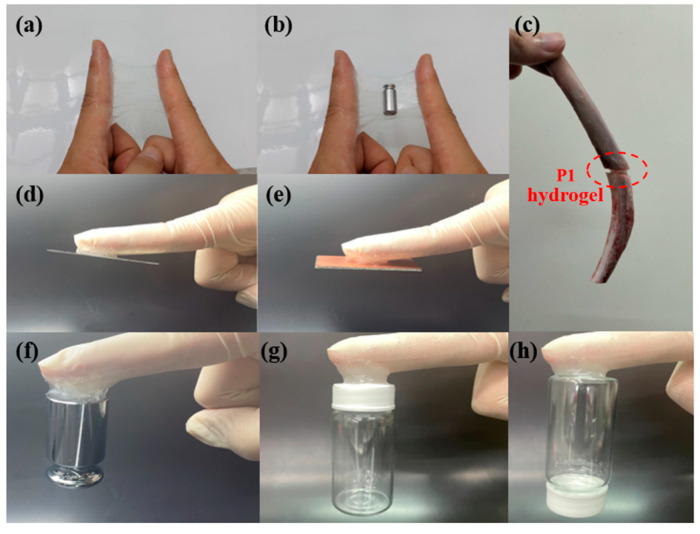
Adhesion images of P1 hydrogel on different substrates: (**a**) skin (during stretching); (**b**) skin (with weights); (**c**) bone; (**d**) Al; (**e**) Cu; (**f**) 100-g steel weight; (**g**) plastic; (**h**) glass.

Subsequently, we tested the adhesion of the P1 hydrogel on various substrates to evaluate its versatility and effectiveness as an adhesive material. As shown in Figure 9a,b, the hydrogel demonstrated excellent adhesion to the skin, which is particularly noteworthy due to the typically challenging nature of achieving strong adhesion on biological tissues. The hydrogel maintained a firm grip on the skin, even under mechanical stress, such as stretching, which is a crucial attribute for applications that require flexibility and durability. This quality makes P1 hydrogel a potential candidate for use in biomedical applications, including wound dressings and wearable sensors, where secure attachment to the skin is essential. In Figure 9a, the hydrogel’s adhesion to the skin is visibly robust, showing minimal signs of detachment even when subjected to forces that would typically compromise adhesion. Moreover, the performance of hydrogel was further tested by applying weights, as shown in Figure 9b. The hydrogel remained securely attached to the skin under the added load, illustrating its capability to withstand additional stress without losing adhesion. This characteristic is particularly important in scenarios where the adhesive must support extra weight or pressure, such as in medical devices or patches that might carry electronic components. Additionally, we explored the potential of the P1 hydrogel as a bone adhesive, as depicted in Figure 9c. The hydrogel successfully bonded two bone fragments together, highlighting its potential application in surgical and orthopedic procedures. This capability suggests that P1 could be used as a bioadhesive in bone repair, offering an alternative to traditional sutures and screws, which can cause additional tissue damage and require removal. Beyond biological tissues, the P1 hydrogel demonstrated strong adhesion to a variety of non-biological substrates, including aluminum (Al) sheets, copper (Cu) sheets, 100-g steel weight, plastic, and glass (Figure 9d–h). This versatility in adhesion is particularly impressive, as it shows the hydrogel’s ability to form strong bonds with both smooth and rough surfaces. Moreover, as shown in Figure S3, we measured the adhesive strength of the P1 hydrogel on Al and Cu sheets, which were 574.6 kPa and 311.1 kPa, respectively. On the other hand, due to the excellent adhesive strength of P1 on glass (717.2 kPa), we conducted repeated adhesion tests. Our experiments revealed that even after five consecutive peel tests, the hydrogel maintained good adhesion (Figure S4). The strong adhesion observed across these different materials suggests that the P1 hydrogel could be useful in a wide range of industrial applications, from electronics to construction, where secure and reliable adhesive materials are required. These comprehensive adhesion tests highlight the P1 hydrogel’s potential as a versatile adhesive material. Its ability to adhere strongly to various substrates, combined with its biocompatibility and mechanical resilience, positions it as a promising candidate for a broad spectrum of applications. Due to its unique properties, the P1 hydrogel has the potential to overcome many limitations of conventional adhesives, offering new opportunities for enhancement and innovation.

## 4. Conclusions

In this work, we developed hydrogels using acrylic acid, acrylamide, carboxymethylcellulose methacrylate, thiol-functionalized polyhedral oligomeric silsesquioxane, and poly(ethylene glycol) dimethacrylate (PEGDM). The hydrogel without PEGDM was designated as P0, while those with PEG monomer molecular weights of 2000, 4000, 8000, 12,000, and 20,000 were labeled as P1, P2, P3, P4, and P5, respectively. It was observed that the P1 hydrogel, with a PEG molecular weight of 2000, achieved an impressive adhesive strength of 717.2 kPa on glass surfaces. Beyond adhesive strength, the P1 hydrogel demonstrated excellent stretchability, support, and fatigue resistance, making it suitable for applications requiring durability under repeated mechanical stress. Furthermore, the development of the P1 hydrogel represents a significant step forward in optimizing the balance between mechanical properties, adhesion, and long-term stability, which has been a challenge in the field of hydrogel research. More importantly, we demonstrated that the P1 hydrogel exhibits excellent biocompatibility and strong adhesion to bone, making it a promising candidate for orthopedic applications as a bone adhesive. Future research could focus on developing adhesive conductive hydrogels to promote tissue repair and regeneration, enable real-time physiological monitoring, and enhance electronic skin functionality. Such hydrogels could have broad applications in biomedical devices, including biosensors, neural interfaces, and cardiac patches, where both strong adhesion and electrical conductivity are essential for seamless integration with biological tissues and long-term stability.

## Data Availability

Data are contained within the article.

## Supplementary Materials

The following supporting information can be downloaded at: https://www.mdpi.com/article/10.3390/polym17050589/s1, Table S1: Mechanical properties of P0–P5 hydrogels; Figure S1: Swelling experiments of P1–P5 hydrogels. P1 is represented in red, P2 in blue, P3 in green, P4 in magenta, and P5 in orange; Table S2: Comparison of reported adhesive strength values of hydrogels on glass; Figure S2: The dissipated energy of the P1 hydrogel: (a) 20 cycles at 200% strain and (b) 15 cycles at 500% strain; Table S3: The dissipated energy of the P1 hydrogel: left for 200% strain and right for 500% strain; Figure S3: The adhesive strength of the P1 hydrogel on Al (black) and Cu (red) sheets; Figure S4: The adhesive strength of the P1 hydrogel on glass tested over five repeated adhesion cycles; Figure S5: ^1^H NMR spectrum of CMC-MA in D_2_O; Figure S6: ^1^H NMR spectrum of POSS-8H in CDCl_3_; Figure S7: ^1^H NMR spectrum of PEGDM in CDCl_3_. References [51,52,53,54,55,56,57,58] are cited in the Supplementary Materials.
