# Supplementary material for: Engineering Hydrogels with Enhanced Adhesive Strength Through Optimization of Poly(Ethylene Glycol) Molecular Weight
Source: Polymers (Basel). 2025 Feb 23;17(5):589. doi: 10.3390/polym17050589 (PMC11902555; doi:10.3390/polym17050589)
Supplement: Supplementary file 1 [file polymers-17-00589-s001.zip › polymers-3467062-supplementary.pdf]

polymers

MDPI

*Supplementary Materials*

# Engineering Hydrogels with Enhanced Adhesive Strength through Optimization of Poly(ethylene glycol) Molecular Weight

**Yin-An Yang [1], Yu-Feng Ni [1], Rajan Deepan Chakravarthy [2], Karl Wu [3,*], Mei-Yu Yeh [1,*] and Hsin-Chieh Lin [2,4,*]**

[1] Department of Chemistry, Chung Yuan Christian University, No. 200, Zhongbei Rd., Zhongli Dist., Taoyuan City 320314, Taiwan, R. O. C.; a0976757006@gmail.com; ricky86831@yahoo.com.tw; myyeh@cycu.edu.tw
[2] Department of Materials Science and Engineering, National Yang Ming Chiao Tung University, Hsinchu 300093, Taiwan, R. O. C.; deepannycu@gmail.com; hclin45@nycu.edu.tw
[3] Department of Orthopaedic Surgery, Far Eastern Memorial Hospital, 21, Section 2, Nanya South Road, New Taipei City 220, Taiwan, R. O. C.; kevinwooo@gmail.com
[4] Center for Intelligent Drug Systems and Smart Bio-Devices (IDS$^2$B), National Yang Ming Chiao Tung University, Hsinchu 30068, Taiwan, R. O. C.
* Correspondence: kevinwooo@gmail.com; myyeh@cycu.edu.tw; hclin45@nycu.edu.tw

Table S1 Mechanical properties of P0-P5 hydrogels.

| Hydrogels | Young's Modulus (kPa) | Tensile Strength (kPa) | Elongation at Break (%) |
|---|---|---|---|
| P0 | 0.6 | 1.4 | 451 |
| P1 | 7.5 | 69.6 | 920 |
| P2 | 7.9 | 73.2 | 918 |
| P3 | 8.7 | 98.9 | 1120 |
| P4 | 5.5 | 65.9 | 1172 |
| P5 | 9.4 | 74.8 | 786 |

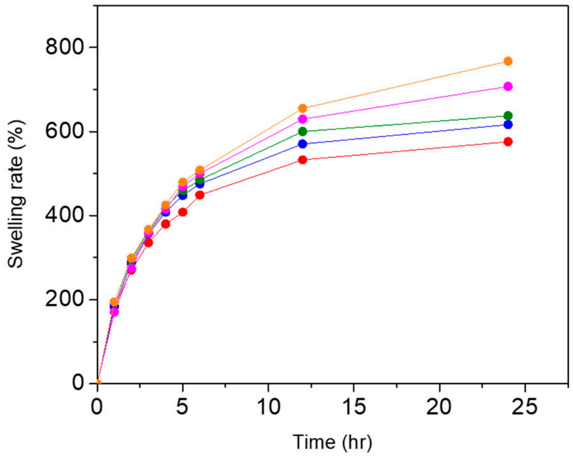


Figure S1: Swelling experiments of P1–P5 hydrogels. P1 is represented in red, P2 in blue, P3 in green, P4 in magenta, and P5 in orange.

Table S2 Comparison of reported adhesive strength values of hydrogels on glass.

| **Component** [a] | **Maximum of adhesive strength (kPa)** | **Reference** |
|---|---|---|
| Aa(Ta)/HMA/AAM | 0.09 | Zhang et al. 2019 |
| PAAM/PAA/GR/PEDOT:PSS | 2.9 | Dong et al 2022 |
| AAM/SA/DA | 5.9 | Chen et al. 2018 |
| PVA/FSWCNT/PDA | 9.2 | Liao et al 2017 |
| PVA/PAA/PEDOT:PSS | 10.97 | Peng et al 2022 |
| AA/$ZnCl_2$/TA | 10.4 | Fu et al. 2021 |
| HACC/PAAM | 14.4 | Wang et al. 2022 |
| TA/Ag/PAA | 35 | Jia et al 2021 |
| This work | 717.2 | / |

[a] Aa: Acrylated adenine (Acrylated thymine); MMA: Hexadecyl methacrylate; AAM: Acrylamide; PAAM: Polyacrylamide; PAA: Poly(acrylic acid); GR: Graphene; PEDOT:PSS: Poly(3,4-ethylenedioxythiophene):poly(styrene sulfonate); SA: Sodium alginate; DA: Dopamine hydrochloride; PVA: Polyvinyl alcohol; FSWCNT: Functionalized single-wall carbon nanotube; PDA: Polydopamine; AA: Acrylic acid; $ZnCl_2$: Zinc chloride; TA: Tannic acid; HACC: Chitosan quaternary ammonium salt.

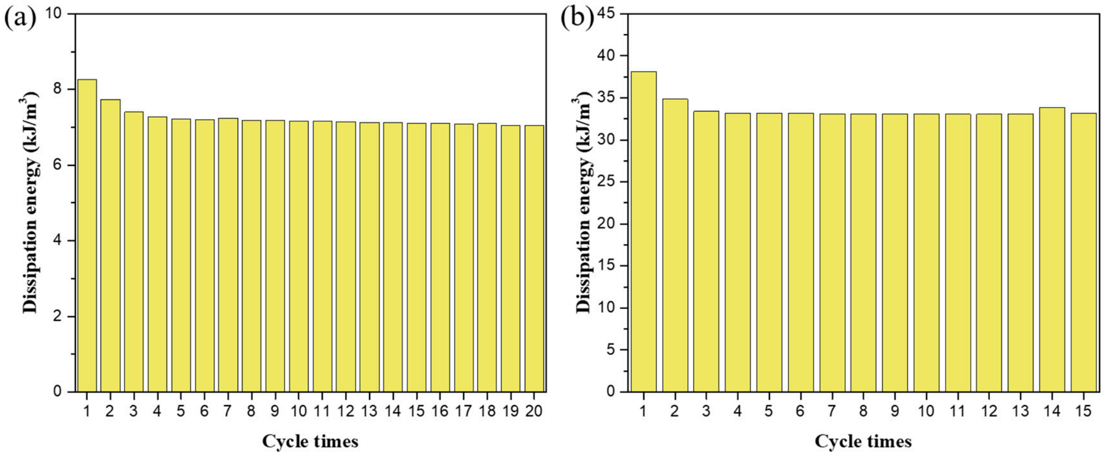


Figure S2: The dissipated energy of the P1 hydrogel: (a) 20 cycles at 200% strain and (b) 15 cycles at 500% strain.

Table S3 The dissipated energy of the P1 hydrogel: left for 200% strain and right for 500% strain.

| **Cycles** | **Dissipated energy (kJ/m$^3$)** | **Retention rate (%)** | **Cycles** | **Dissipated energy (kJ/m$^3$)** | **Retention rate (%)** |
|---|---|---|---|---|---|
| 1 | 8.259 | / | 1 | 38.884 | / |
| 2 | 7.733 | 100 | 2 | 34.850 | 100 |
| 3 | 7.398 | 95.7 | 3 | 33.398 | 95.8 |
| 4 | 7.277 | 94.1 | 4 | 33.174 | 95.2 |
| 5 | 7.217 | 93.3 | 5 | 33.158 | 95.1 |
| 6 | 7.204 | 93.2 | 6 | 33.143 | 95.1 |
| 7 | 7.242 | 93.7 | 7 | 33.108 | 95.0 |
| 8 | 7.175 | 92.8 | 8 | 33.090 | 94.9 |
| 9 | 7.174 | 92.8 | 9 | 33.048 | 94.8 |
| 10 | 7.158 | 92.6 | 10 | 33.061 | 94.9 |
| 11 | 7.156 | 92.5 | 11 | 33.054 | 94.8 |
| 12 | 7.143 | 92.4 | 12 | 33.045 | 94.8 |
| 13 | 7.128 | 92.2 | 13 | 33.074 | 94.9 |
| 14 | 7.112 | 92.0 | 14 | 33.812 | 97.0 |
| 15 | 7.109 | 91.9 | 15 | 33.156 | 95.1 |
| 16 | 7.105 | 91.9 | | | |
| 17 | 7.091 | 91.7 | | | |
| 18 | 7.094 | 91.7 | | | |
| 19 | 7.045 | 91.1 | | | |
| 20 | 7.042 | 91.1 | | | |

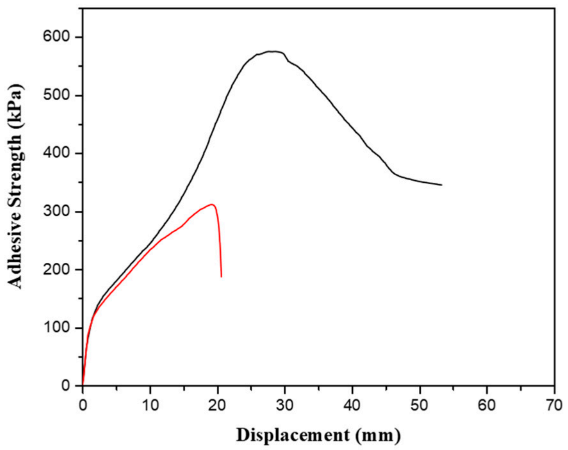


Figure S3: The adhesive strength of the P1 hydrogel on Al (black) and Cu (red) sheets.

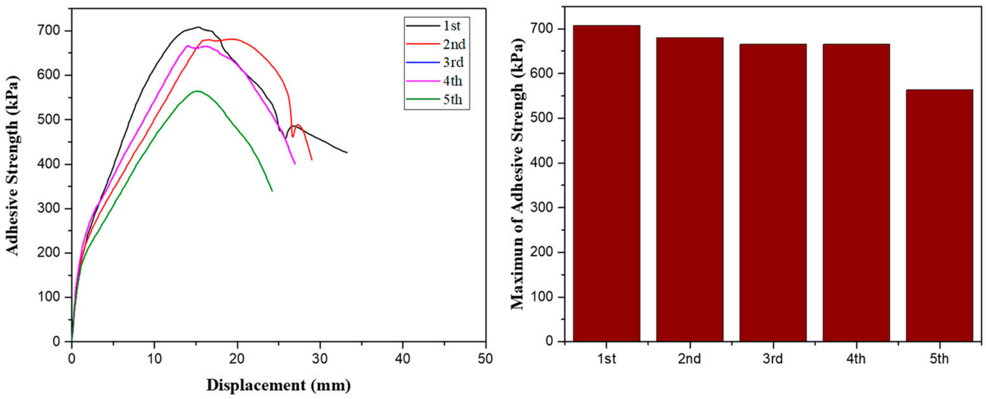


Figure S4: The adhesive strength of the P1 hydrogel on glass was tested over five repeated adhesion cycles.

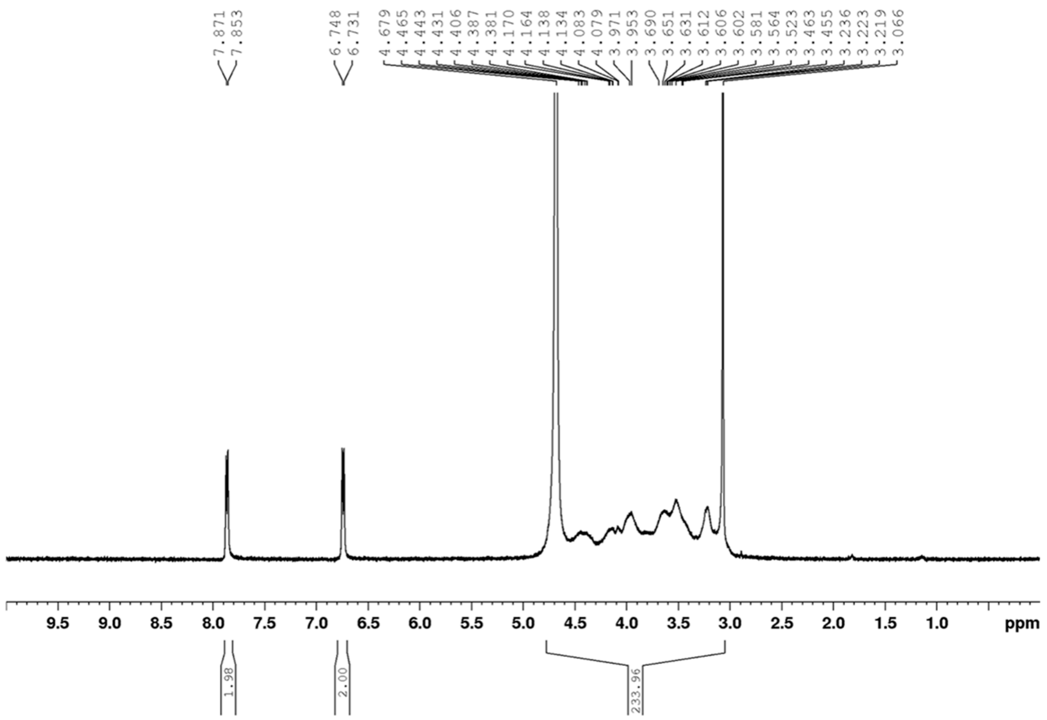


Figure S5: $^{1}$H NMR spectrum of CMC-MA in $D_2O$.

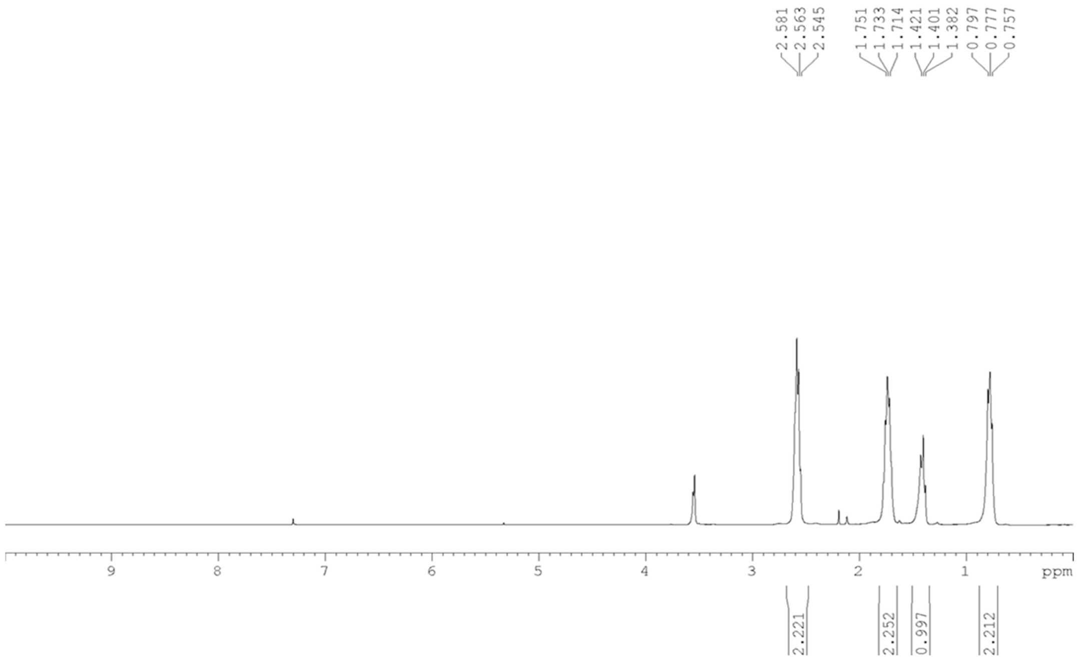


Figure S6: $^{1}$H NMR spectrum of POSS-8H in $CDCl_3$.

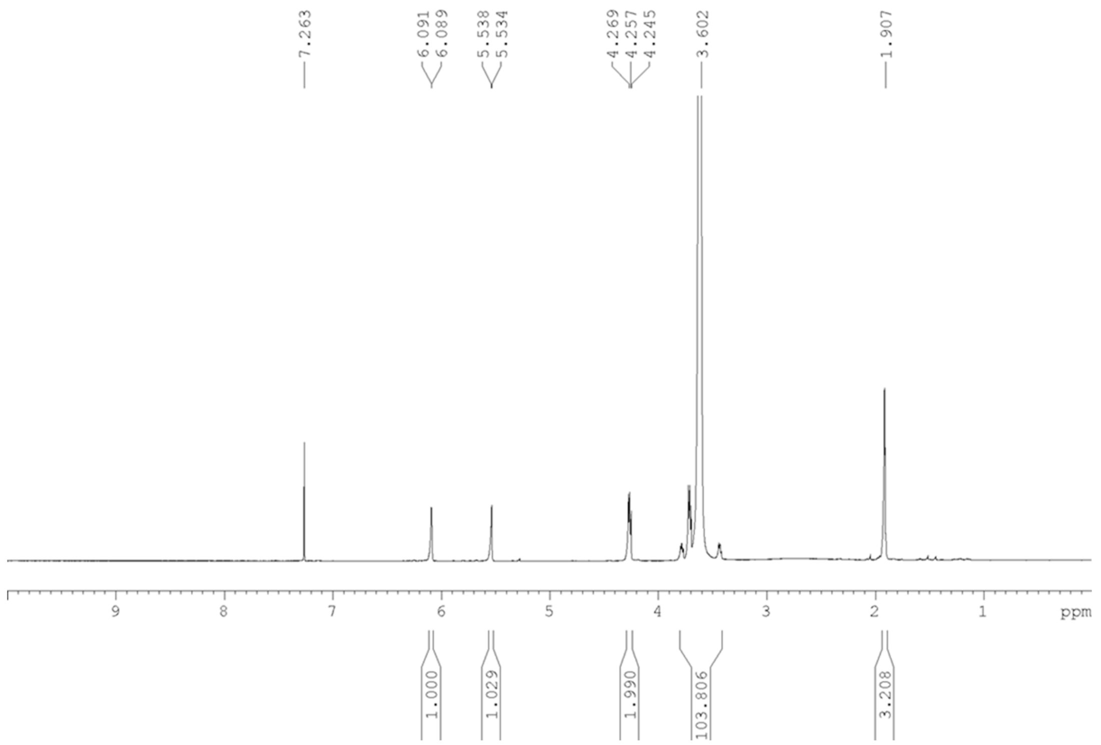


Figure S7: $^1$H NMR spectrum of PEGDM in $CDCl_3$.

Reference

Chen, T.; Chen, Y.; Rehman, H. U.; Chen, Z.; Yang, Z.; Wang, M.; Li, H.; Liu, H. Ultratough, Self-Healing, and Tissue-Adhesive Hydrogel for Wound Dressing. ACS Appl. Mater. Interfaces 2018, 10, 33523-33531.

Dong, L.; Wang, M.; Wu, J.; Zhu, C.; Shi, J.; Morikawa, H. Stretchable, Adhesive, Self-Healable, and Conductive Hydrogel-Based Deformable Triboelectric Nanogenerator for Energy Harvesting and Human Motion Sensing. ACS Appl. Mater. Interfaces 2022, 14, 9126-9137.

Fu, Q.; Hao, S.; Meng, L.; Xu, F.; Yang, J. Engineering Self-Adhesive Polyzwitterionic Hydrogel Electrolytes for Flexible Zinc-Ion Hybrid Capacitors with Superior Low-Temperature Adaptability. ACS Nano 2021, 15, 18469-18482.

Jia, Z.; Lv, X.; Hou, Y.; Wang, K.; Ren, F.; Xu, D.; Wang, Q.; Fan, K.; Xie, C.; Lu, X. Mussel-inspired nanozyme catalyzed conductive and self-setting hydrogel for adhesive and antibacterial bioelectronics. Bioact. Mater. 2021, 6, 2676-2687.

Liao, M.; Wan, P.; Wen, J.; Gong, M.; Wu, X.; Wang, Y.; Shi, R.; Zhang, L. Wearable, Healable, and Adhesive Epidermal Sensors Assembled from Mussel-Inspired Conductive Hybrid Hydrogel Framework. Adv. Funct. Mater. 2017, 27, 1703852.

Peng, X.; Wang, W.; Yang, W.; Chen, J.; Peng, Q.; Wang, T.; Yang, D.; Wang, J.; Zhang, H.; Zeng, H. Stretchable, compressible, and conductive hydrogel for sensitive wearable soft sensors. J. Colloid Interface Sci. 2022, 618, 111-120.

Wang, L.; Zhou, M.; Xu, T.; Zhang, X. Multifunctional hydrogel as wound dressing for intelligent wound monitoring. Chem. Eng. J. 2022, 433, 134625.

Zhang, Q.; Liu, X.; Duan, L.; Gao, G. Ultra-stretchable wearable strain sensors based on skin-inspired adhesive, tough and conductive hydrogels. Chem. Eng. J. 2019, 365, 10-19.
